# Serum soluble ST2 is associated with ER-positive breast cancer

**DOI:** 10.1186/1471-2407-14-198

**Published:** 2014-03-18

**Authors:** Da-peng Lu, Xiang-yu Zhou, Lu-tian Yao, Cai-gang Liu, Wei Ma, Feng Jin, Yun-fei Wu

**Affiliations:** 1 Department of Breast Surgery, First Affiliated Hospital of China Medical University, 155 Nanjing North Street, Heping District, Shenyang 110001, PR China; 2 Department of Hepatobiliary Surgery, First Affiliated Hospital of China Medical University, Shenyang 110001, China; 3 Department of Oncology Surgery, First Affiliated Hospital of China Medical University, Shenyang 110001, China

**Keywords:** Soluble ST2, sST2, Interleukin-33, IL-33, Vascular endothelial growth factor, VEGF, Breast cancer

## Abstract

**Background:**

ST2, a member of the interleukin (IL)-1receptor family, regulates Th1/Th2 immune responses in autoimmune and inflammatory conditions. However, the role of ST2 signaling in tumor growth and metastasis of breast cancers has not been investigated. This study investigated the possible role of soluble ST2 (sST2) in breast cancer.

**Methods:**

The serum levels of IL-33, sST2, and vascular endothelial growth factor (VEGF) in 150 breast cancer patients and 90 healthy women were measured by enzyme-linked immunosorbent assay. Estrogen receptor(ER), progesterone receptor, human epithelial receptor (HER)-2, and cell cycle regulated protein Ki-67 were measured. Clinical stage, tumor size, lymph node metastasis, and histological type were also recorded.

**Results:**

The serum levels of sST2, IL-33, and VEGF were significantly higher in breast cancer patients than in the control group (*P* < 0.05, each). Serum sST2 levels in ER-positive breast cancer patients were significantly associated with age, histological type, clinical stage, tumor size, and Ki-67 status (*P* < 0.05, each). Moreover, the serum levels of IL-33 and sST2 in breast cancers significantly correlated with VEGF levels (IL-33: *r* = 0.375, *P* < 0.0001; sST2: *r* = 0.164, *P* = 0.045). Serum levels of sST2, IL-33, and VEGF decreased after modified radical mastectomy in ER-positive breast cancers. Serum levels of IL-33, sST2, and VEGF and clinicopathological factors were not significantly correlated with disease-free survival and overall survival of ER-positive breast cancer women during follow-up.

**Conclusion:**

Serum sST2 levels in ER-positive breast cancer patients are significantly associated with factors that indicate poor prognosis.

## Background

Breast cancer is one of the most common malignancies and the leading cause of mortality in women in western countries and in China [1]. Thus, early diagnosis and effective therapies for breast cancer are imperative. Differential biomarkers are an effective diagnostic method for screening and targeting breast cancer. However, a limited number of biomarkers for breast cancer have been validated for clinical application [2].

ST2 is a member of the interleukin 1 receptor (IL-1R) family that was originally identified in oncogene or serum-stimulated fibroblasts [3]. Differential mRNA processing within the ST2 gene generates three isoforms: a soluble form, a membrane-anchored form, and a variant ST2. The soluble ST2 (sST2) is especially found in embryonic tissues, and is secreted by macrophages, type 2 helper T (Th2) cells, and fibroblasts [4]. IL-33, as ST2’s natural ligand which is expressed in many tissues, can induce the secretion of both proinflammatory and anti-inflammatory cytokines [5]. IL-33 induces angiogenesis and vascular permeability through ST2 and multiple inflammatory angiogenic factors such as vascular endothelial growth factor (VEGF) [6]. IL-33/ST2 signaling has a protective role against parasites, in atherosclerosis, and obesity, but it can enhance Th2 and mast cell-mediated diseases such as asthma and anaphylaxis [7]. Schmieder et al. [8] found that IL-33 might act as a crucial mediator in inflammation-associated pancreatic carcinogenesis. Jovanovic et al. [9] reported that the mice lacking ST2 showed slower breast cancer growth and progression than normal mice. However, the role of ST2 in tumor growth and metastasis in breast cancer patients has not been explored.

In the present study, we investigated whether serum IL-33 or sST2 correlated with VEGF levels or clinicopathological features in breast cancer tissues.

## Methods

### Patients

The Ethics Committee at First Affiliated Hospital of China Medical University approved the study, in accordance with the Declaration of Helsinki. All individuals gave written informed consent for participation in the study.

Between 2011 and 2013 at First Affiliated Hospital of China Medical University, we prospectively recruited 150 women aged 51.7 ± 8.4y with high risk of breast cancer determined by imaging diagnosis. The control group consisted of 90 healthy women aged 50.2 ± 8.5y whose ages were matched to the patient cohort. The breast cancers met updated criteria for metastatic breast cancer established by the National Comprehensive Cancer Network [10]. The 150 patients had never undergone mastectomy, breast-conserving surgery, or anticancer medication for breast cancer. Serum samples were obtained 1–2 weeks before surgery. Twenty-six patients who underwent modified radical mastectomy and were not using anticancer medication were selected 4-6 weeks after surgery based on estrogen receptor (ER)-positive expression.

Patients were followed up at an interval of 12–24 weeks, with the day pathological diagnosis was performed considered as the first day of follow-up. The 150 patients had been followed up for a median of 73 weeks (range, 24–121 weeks), during which time there were 9 relapses and 4 deaths. Disease-free survival (DFS) was defined as the time between surgery and the date of first local recurrence, distant metastasis, second primary cancer of another organ, or death from any cause whichever appeared first during follow-up. Patients known to be alive with no evidence of disease were censored at the last follow-up date. Overall survival (OS) was defined from surgery to death for any cause, and patients who were alive were censored at date of last follow-up visit.

The following information was recorded: tumor size, axillary lymphnode, cancers stage according to the tumor-node-metastasis classification, histological type, status of ER and progesterone receptor (PR), human epithelial receptor (HER)-2 protein status and cell cycle regulated protein Ki-67. All patients’ serum samples were stored at–80°C before use.

### Assay for serum levels of sST2, IL-33, and VEGF

Serum levels of IL-33, sST2, and VEGF were measured using enzyme-linked immunosorbent assay in accordance with the manufacturer’s directions (R&D systems, Minneapolis, MN, USA). The sensitivities of the immunoassays for IL-33 and sST2/VEGF were 33 pg/mL and 23 pg/mL, respectively.

### Statistical analysis

All analyses were performed using SPSS18.0 (SPSS, Chicago, IL) and GraphPad5 software. The data are presented as the mean ± standard error of the mean. Continuous variables from the study were analyzed by the ANOVA and/or the Student’s t test with a parametric distribution or the Mann–Whitney U test with a nonparametric distribution. Spearman’s correlation coefficient was used to test the correlations between two variables. Disease-free survival and overall survival were estimated using the Kaplan–Meier method and Cox regression analyses. The hazard ratios and corresponding 95% confidence intervals (CIs) were calculated with Cox’s proportional hazards model. A *P* < 0.05 was considered significant.

## Results

### Serum levels of sST2, IL-33 and VEGF in breast cancer patients

**Figure 1:**
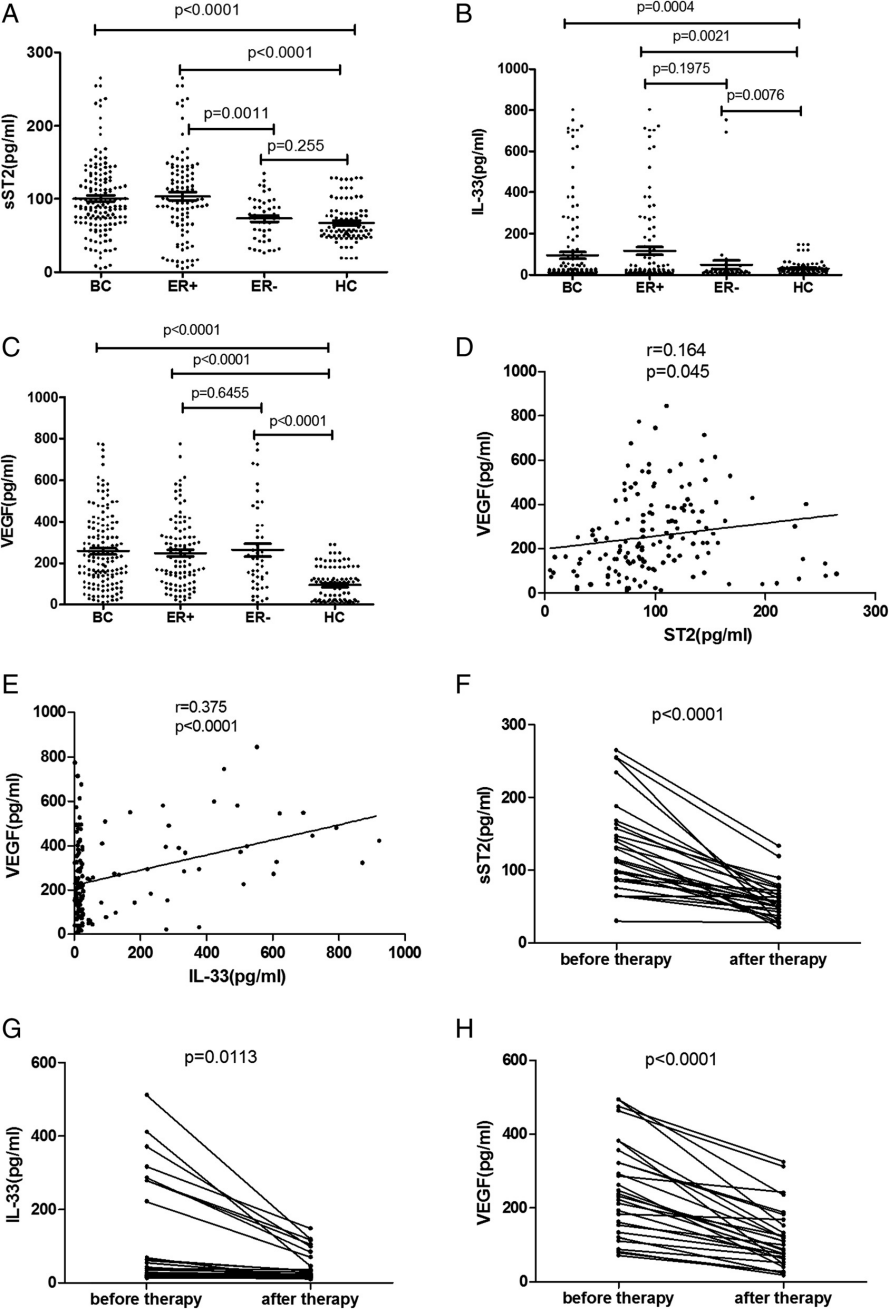
**Analysis of the serum levels of sST2, IL-33 and VEGF in breast cancer patients. A**. The serum levels of sST2 in breast cancer patients and the prevalence of ER-positive breast cancer patients (103/150, 68.7%) were significantly higher than those of the control group (P < 0.05, respectively). **B**. The serum levels of IL-33 in all breast cancer patients taken together, ER-positive breast cancer patients, and ER-negative breast cancer patients were significantly higher than those of the control group (all p < 0.05). **C**. The serum levels of VEGF in all breast cancer patients taken together, ER-positive breast cancer patients, and ER-negative breast cancer patients were significantly higher than those of the control group (all p < 0.05). **D**. The serum levels of sST2 in breast cancer patients had significant correlations with VEGF (r = 0.164, P = 0.045). **E**. The serum levels of IL-33 in breast cancer patients had significant correlations with VEGF (r = 0.375, P < 0.0001). **F**. The serum levels of sST2 significantly decreased after modified radical mastectomy (P < 0.0001). **G**. The serum levels of IL-33 significantly decreased after modified radical mastectomy (P = 0.0113). **H**. Serum levels of VEGF decreased after therapy (P < 0.0001).Abbreviations: BC=breast cancer, HC=healthy controls, ER=estrogen receptor.

The serum levels of sST2 in breast cancer patients (n = 150) and the prevalence of ER-positive breast cancer patients (103/150, 68.7%) were significantly higher than those of the control group (100.5 ± 4.1 pg/mL compared with 67.6 ± 2.9 pg/mL, *P* < 0.0001; 103.6 ± 5.8 pg/ml compared with 67.6 ± 2.9 pg/ml, *P* < 0.0001; Figure 1A), but sST2 levels in ER-negative breast cancer patients (47/150, 31.3%) was not significantly different from those of the healthy controls (73.3 ± 4.1 pg/mL compared with 67.6 ± 2.9 pg/mL, *P* = 0.2583; Figure 1A). sST2 levels in ER-positive breast cancer patients were significantly higher than in ER-negative patients (103.6 ± 5.8 pg/mL compared with 73.3 ± 4.1 pg/mL, *P* = 0.0011; Figure 1A). In addition, serum sST2 levels in ER-positive breast cancer patients were bifurcated at mean value (103.6 pg/mL).Values of >103.6 pg/mL indicated high levels of serum sST2 in ER-positive breast cancer patients, and values of <103.6 pg/mL indicated low levels of serum sST2.

The serum levels of IL-33 and VEGF in all breast cancer patients taken together, ER-positive breast cancer patients, and ER-negative breast cancer patients were significantly higher than those of the control group (IL-33: 105.5 ± 16.0 pg/mL, 162.0 ± 39.2 pg/mL, 306.0 ± 140.8 pg/mL compared with 30.7 ± 3.3 pg/mL *P* < 0.05,each; Figure 1B; VEGF: 257.6 ± 14.6 pg/mL, 248.5 ± 16.5 pg/mL, 263.1 ± 29.8 pg/mL compared with 93.0 ± 8.6 pg/mL *P* < 0.05,each; Figure 1C). IL-33 and VEGF levels in ER-positive breast cancer patients were not significantly different than those of the ER-negative patients (IL-33: 162.0 ± 39.2 pg/mL compared with 306.0 ± 140.8 pg/mL, *P* = 0.1973, Figure 1AB; VEGF: 248.5 ± 263.1 ± 29.8 pg/mL compared with 306.0 ± 140.8 pg/mL, *P* = 0.6455, Figure 1C). In addition, serum IL-33/VEGF levels in ER-positive breast cancer patients were bifurcated at mean value (IL-33: 162.0 pg/mL; VEGF: 248.5 pg/mL). Values of >162.0 pg/mL indicated high levels of serum IL-33 in ER-positive breast cancer patients, and values of <162.0 pg/mL indicated low levels of serum IL-33. Values of >248.5 pg/mL indicated high levels of serum VEGF in ER-positive breast cancer patients, and values of < thinsp;248.5 pg/mL indicated low levels of serum VEGF.

### Association of sST2, IL-33 and VEGF expression levels and clinical-pathological factors in ER-positive breast cancer

Serum levels of sST2 in invasive breast cancer patients (n = 91) were significantly higher than those of breast cancer patients with ductal carcinoma *in situ* (DCIS, n = 12; 108.1 ± 6.3 pg/mL compared with 69.0 ± 11.5 pg/mL, *P* = 0.0307; Table 1). Serum levels of sST2 increased with increasing tumor size: serum sST2 concentrations of Tis (n =12), T1 (≤2 cm, n = 37), T2 (>2 cm, ≤5 cm, n = 50), and T3(>5 cm, n = 4) tumors were 69.0 ± 11.5 pg/mL, 96.9 ± 9.2 pg/mL, 116.8 ± 9.1 pg/mL, and 123.5 ± 48.9 pg/mL, respectively. The sST2 serum levels of patients with T2 tumors was significantly higher than those of patients with Tis tumors (*P* = 0.0165; Table 1).

**Table 1:** Clinicopathological implication of sST2 levels in ER-positive breast cancers

**Parameter**	**N (total = 103)**	**sST2-2(pg/ml)**	
		**Mean ± SEM**	***p-*** **value**
Age (years)			
<50 years old	45	75.7 ± 6.6	<0.0001*
≥50 years old	58	125.2 ± 7.9	
Histological type			
IBC	91	108.1 ± 6.3	0.0307*
DCIS	12	69.0 ± 11.5	
Stage			
0	12	69.0 ± 11.5	0.0108*
I	27	96.5 ± 11.8	-
II	49	112.4 ± 9.0	-
III	15	115.0 ± 11.8	
Tumor size (cm)			
Tis	12	69.0 ± 11.5	0.0165*
T1(≤2)	37	96.9 ± 9.2	-
T2 (>2, ≤5)	50	116.8 ± 9.1	
T3 (>5)	4	123.5 ± 8.6	-
Lymphonodus status			
Positive	70	97.3 ± 7.2	0.0119
Negative	33	116.8 ± 9.6	
PR status			
Positive	99	102.6 ± 6.0	0.4439
Negative	4	125.9 ± 16.6	
Tissue Her-2			
Positive	10	109.9 ± 13.5	0.7252
Negative	93	102.9 ± 6.3	
Ki67 status			
>14%(+)	74	120.5 ± 6.6	<0.0001*
≤14% (+)	29	60.4 ± 7.7	

*Abbreviation:* No = number, IBC = invasive breast cancer, T = tumor size, Tis: tumor in situ, DCIS: ductal carcinoma in situ, ER **=** estrogen receptor, PR = progesterone receptor, Her-2 = human epithelial receptor-2.

*: *p <* 0.05.

Numerical data are shown as mean ± standard error *P* < 0.05, indicated a significant association.

Serum levels of sST2 increased with increasing stage of breast cancer. The serum sST2 concentrations of stage0 (n = 12), stage I (n = 27), stage II (n = 49), and stage III (n = 15) were 69.0 ± 11.5 pg/mL, 96.5 ± 11.8 pg/mL, 112.4 ± 9.0 pg/mL, and115.0 ± 11.8 pg/mL, respectively. Serum sST2 levels correlated with stage of ER-positive breast cancer, with significant differences between stages 0 and III (*P* = 0.0108) and between stages II and 0 (*P* = 0.0275; Table 1).

The serum sST2 levels in ER-positive breast cancer patients whose age was ≥50y old (125.2. ± 7.9 pg/mL) were significantly higher than those of younger ER-positive patients (75.7 ± 6.6 pg/mL, *P* < 0.0001; Table 1).The serum concentrations of sST2 in ER-positive breast cancer patients were not correlated with lymphonodus status (*P* > 0.05; Table 1).The serum concentrations of IL-33 and VEGF in breast cancer patients were not correlated with age, histological type, tumor size, lymphonodus status, or stage (*P* > 0.05).

### Associations between serums ST2 and PR, Her-2, and Ki-67 in ER-positive breast cancers

Among ER-positive breast cancer patients, those with high Ki-67 expression (>14%[+]) had significantly higher serum sST2 levels than did those with low-Ki-67 expression (120.5 ± 6.6 pg/mL compared with 60.4 ± 7.7 pg/mL, *P* < 0.0001, Table 1). However, among these patients there were no significant differences in PR status or Her-2 status (both *P* > 0.05; Table 1).

### Correlations of serum levels of sST2/IL-33 and VEGF

The serum levels of sST2 and IL-33 in breast cancer patients had significant correlations with VEGF (sST2: *r* = 0.164, *P* = 0.045; Figure 1D, IL-33: *r* = 0.375, *P* < 0.0001; Figure 1E).

### Serum levels of IL-33, sST2, and VEGF decreased after modified radical mastectomy in patients with ER-positive breast cancer

Among the 150 patients, 26 ER-positive breast cancer patients under went modified radical mastectomy. The serum levels of both IL-33 and sST2 significantly decreased after modified radical mastectomy (sST2: *P* < 0.0001, Figure 1F; IL-33: *P* = 0.0113, Figure 1G). Serum levels of VEGF also decreased after therapy (*P* < 0.0001; Figure 1H).

### Serum levels of IL-33, sST2, and VEGF and clinicopathological factors associated with disease-free survival and overall survival in patients with ER-positive breast cancer

The 103 patients with ER-positive breast cancer had been followed up for a median of 73 weeks (range, 24–121 weeks), during which time there were 7 relapses and 3 deaths. Table 2 showed that serum sST2, IL-33 and VEGF levels were not correlated with disease-free survival of ER-positive breast cancer women during follow-up (high versus low) (sST2: HR, 2.371; 95% CI, 0.520-10.812; P = 0.265; IL-33: HR, 0.988; 95% CI, 0.190-5.126; P = 0.988; VEGF: HR, 1.308; 95% CI, 0.119-14.437; P = 0.826). Serum sST2, IL-33 and VEGF levels were not correlated with overall survival of ER-positive breast cancer women during follow-up (high versus low) (sST2: HR, 147.956; 95% CI, 0.006-3.743E5; P = 0.334; IL-33: HR, 0.870; 95% CI, 0.078-9.696; P = 0.910; VEGF: HR, 0.516; 95% CI, 0.115-2.309; P = 0.387; Table 2). All clinicopathological factors were not correlated with disease-free survival and overall survival of ER-positive breast cancer women during follow-up (all p > 0.05; Table 2).

**Table 2:** Clinicopathological factors associated with disease-free survival and overall survival in patients with ER-positive breast cancer

**Parameter**	**N(total = 103)**	**Disease-free survival**			**Overall survival**		
		**Hazard ratio**	**(95% CI)**	**P-value**	**Hazard ratio**	**(95% CI)**	**P-value**
Age (years)							
<50 years old	45	1			1		
≥50 years old	58	1.352	0.262-6.976	0.719	0.275	0.025-3.037	0.292
Histological type							
IBC	91	1			1		
DCIS	12	0.044	0.000-1.173E6	0.721	0.044	0.000-2.452E12	0.846
Stage							
0, I and II	88	1			1		
III	15	1.782	0.345-9.194	0.490	2.218	0.201-24.536	0.516
Tumor size (cm)							
≤2 cm	49	1			1		
>2 cm	54	0.460	0.096-2.196	0.330	0.136	0.011-1.636	0.116
Lymphonodus status							
Positive	70	1			1		
Negative	33	2.834	0.539-14.891	0.219	2.366	0.205-27.247	0.490
PR status							
Positive	99	1			1		
Negative	4	0.046	0.000-6.748E5	0.715	0.046	0.000-9.501E8	0.799
Tissue Her-2							
Positive	10	1			1		
Negative	93	31.064	0.011-8.581E4	0.395	32.686	0.000-3.286E6	0.533
Ki67 status							
>14%(+)	74	1			1		
≤14% (+)	29	1.229	0.238-6.339	0.805	1.506	0.0136-16.627	0.738
sST2 status							
High	48	1			1		
Low	55	2.371	0.520-10.812	0.265	147.956	0.006-3.743E5	0.334
IL-33 status							
High	23	1			1		
Low	80	0.988	0.190-5.126	0.988	0.870	0.078-9.696	0.910
VEGF status							
High	47	1			1		
Low	56	1.308	0.119-14.437	0.826	0.516	0.115-2.309	0.387

*Abbreviation:* No = number, IBC = invasive breast cancer, T = tumor size, Tis: tumor in situ, DCIS: ductal carcinoma in situ, ER **=** estrogen receptor, PR = progesterone receptor, Her-2 = human epithelial receptor-2, CI = Confidence interval.

*P* < 0.05, indicated a significant association.

## Discussion

In the present study, we investigated whether serum IL-33 or sST2 correlated with VEGF levels or clinicopathological features in breast cancer tissues. We used an enzyme-linked immunosorbent assay to measure serum levels of IL-33, sST2, and VEGF. Significant associations were found between sST2 and factors that indicate poor prognosis in ER-positive breast cancer.

We found that serum levels of IL-33 and sST2 in breast cancer patients were significantly higher than in healthy women. There are other evidences that IL-33 and sST2 may be involved in the pathogenesis of cancer. Gillibert et al. [11] showed that serum levels of sST2 in metastatic breast cancer patients were higher than in primary breast cancer patients, and our study showed serum levels of sST2 in primary breast cancer patients were significantly higher than in healthy women. That demonstrated high sST2 level might be a risk factor for breast cancer. According to Küchler et al. [12], IL-33 was generally expressed in the nuclei of resting endothelia, but was rapidly down regulated in tumors. sST2 functions as an antagonistic decoy receptor which serves as a ligand sink by competing for IL-33 with membrane-bound ST2 [5]. Therefore, it is not surprising that elevated levels of IL-33 are accompanied by an sST2 upregulation.

Our study also found the serum levels of sST2 in ER-positive breast cancer patients were significantly higher than those of the control group, but sST2 levels in ER-negative breast cancer patients were not significantly different from those of healthy controls. ER is recognized as an independent prognostic factor in breast cancer, and recent studies have shown survival advantages among women with ER-positive tumors relative to women with ER-negative tumors [13]. Therefore, we evaluated the sST2 serum levels in ER-positive subgroups. Among these subgroups, serum levels of sST2 in invasive breast cancer patients were significantly higher than those of DCIS patients, and there was a significant correlation with age, tumor size, and clinical stage. Our study found sST2 levels in ER-positive breast cancer patients did not correlate with the Her-2 status confirms previous results by Gillibert-Duplantier et al. [11]. We held same reason for limiting cases (Her-2 positive patients: 10/103; Table 1). We should enlarge sample size regarding breast cancer patients in the future study. We also found that serum sST2 levels in ER-positive breast cancer patients with high-Ki-67 were significantly higher than those of the low-Ki-67 group. Sahin et al. [14] demonstrated a strong correlation between the percentage of cells positive for Ki-67 and nuclear grade, age, and mitotic rate in breast carcinomas. Therefore, we surmise that sST2 may also be significantly associated with factors that indicate poor prognosis in ER-positive breast cancer.

Angiogenesis is inevitable in tumor growth and the formation of locoregional and systemic metastases. The role of VEGF in angiogenesis is well recognized, and a high expression of VEGF indicates in most cases a poor prognosis [15]; VEGF expressed by endothelial and tumor cells could be considered a promising target to limit both tumor growth and angiogenesis. There are several contradictory results reported in the literature on serum levels of VEGF in breast cancer patients. While Heer K et al. [16] demonstrated elevated VEGF serum levels in cancer patients compared to healthy controls and the correlation between a high concentration of VEGF, tumor size, and metastasis to regional lymph nodes, Hodorowicz-Zaniewska et al. [17] did not. Moreover, in the latter study VEGF levels did not correlate with clinicopathological factors. In our study, we found the serum levels of VEGF were significantly higher in breast cancer patients than in the control group, but not correlated with clinicopathological factors. The composition of the study cohort obviously determines the quality of the outcome measure, which needs us to enlarge sample size regarding breast cancer patients in the future study, limiting false-positive conclusion due to random errors. The present study found for the first time that serum levels of IL-33 and sST2 in breast cancer patients had significant correlations with VEGF. We also found serum levels of IL-33, sST2, and VEGF decreased after modified radical mastectomy in patients with ER-positive breast cancer. Whether IL-33, sST2, and VEGF play a role in the promotion or eradication of tumors, the tumor immunoregulatory effect on them is weakened along with tumor disappearance.

The present study evaluated the correlation between serum sST2 and IL-33 levels and ER-positive breast cancer patient survival for the first time. We found that serum levels of IL-33, sST2, and VEGF and clinicopathological factors were not correlated with disease-free survival and overall survival of ER-positive breast cancer women during follow-up. But the results may not explain this problem completely because of short follow-up time (a median of 73 weeks (range, 24–121 weeks)). We should continue follow-up to give a demonstration.

From the above mentioned evidence, sST2 may imply a therapeutic target for ER-positive breast cancer. One study reported that deletion of ST2 signaling enhanced the antitumor immune response in a murine model of metastatic breast carcinoma [18]. There is a need to conduct more researches in this field to give a demonstration.

Some limitations of the current study should be acknowledged. Firstly, we did not evaluate the interactions between serum sST2 levels and other therapies (e.g., anticancer medication and endocrine treatment), resulting in a lack of information showing the value of serum sST2 concentration in evaluating effectiveness of those therapies. Secondly, relatively small sample size regarding breast cancer patients, making the results susceptible to false-positive conclusion due to random errors. Thirdly, short follow-up time may make the survival data inaccuracy. In addition, we did not explore sST2 level after anticancer medication or endocrine treatment in detail, which results in uncertainty on this issue and calls for more studies.

Despite limitations, the current study revealed sST2 involved in the pathogenesis of ER-positive breast cancer.

## Conclusion

Serum sST2 levels are higher in ER-positive breast cancer patients, and are significantly associated with factors that indicate poor prognosis.

## Abbreviations

DICS: Ductal carcinoma in situ; ER: Estrogen receptor; HER-2: Human epithelial receptor2; PR: Progesterone receptor; sST2: Soluble ST2; VEGF: Vascular endothelial growth factor.

## Competing interests

None of the authors have any actual or potential conflicts of interest with other people or organizations within three year of initiating the work presented here.

## Authors’ contributions

All authors made substantial contributions to the conception and design of the study, and acquisition, analysis, and interpretation of the data. All authors were involved in drafting the manuscript or revising it, and all read and approved the final manuscript.

## Pre-publication history

The pre-publication history for this paper can be accessed here:

http://www.biomedcentral.com/1471-2407/14/198/prepub
